# Physicians' Perspective on Prescribing Patterns and Knowledge on Antimicrobial Use and Resistance in Penang, Malaysia: A Qualitative Study

**DOI:** 10.3389/fpubh.2020.601961

**Published:** 2020-11-25

**Authors:** Ali Akhtar, Amer Hayat Khan, Hadzliana Zainal, Mohamed Azmi Ahmad Hassali, Irfhan Ali, Long Chiau Ming

**Affiliations:** ^1^Discipline of Clinical Pharmacy, School of Pharmaceutical Sciences, Universiti Sains Malaysia, George Town, Malaysia; ^2^Discipline of Social and Administrative Pharmacy, School of Pharmaceutical Sciences, Universiti Sains Malaysia, George Town, Malaysia; ^3^Senior Consultant, Hospital Pulau Pinang, George Town, Malaysia; ^4^Pengiran Anak Puteri Rashidah Sa'adatul Bolkiah (PAPRSB) Institute of Health Sciences, Universiti Brunei Darussalam, Gadong, Brunei Darussalam

**Keywords:** antimicrobials, interviews, Malaysia, physicians, qualitative study, resistance

## Abstract

**Background:** Unnecessary antimicrobial use is an emerging problem throughout the world. To design future interventions to ensure rational antimicrobial use and decrease the risk of antimicrobial resistance, physician's knowledge and prescribing practices of antimicrobials should be assessed. Therefore, the main objective of this study is to investigate the physician's knowledge along with their prescribing patterns of antimicrobials in their health care system.

**Methods:** The present qualitative study was conducted in a tertiary care public hospital located at Penang island, situated in Northwest of Malaysia. A total of 12 semi-structured, face to face interviews were conducted with purposive sampling technique. Physicians recruited had different specialties. All interviews were audio recorded, then transcribed into English language and analyze by thematic content analysis.

**Results:** Four major themes were identified: (1) prescribing patterns of physicians regarding antimicrobials; (2) physician's knowledge about antimicrobials; (3) antimicrobial resistance; (4) satisfaction with management of infections. Physicians believed in regular educational activities and updates about the latest antimicrobial guidelines may change the prescribing behavior of physicians to optimize the use of antimicrobials. This may lead to decrease in burden of antimicrobial resistance in their health care system. Physicians emphasized that stricter rules and regular monitoring of antimicrobial use should be implemented to overcome the main challenges of antimicrobial resistance.

**Conclusion:** Different factors were identified to assist optimized use of antimicrobials and decrease the risk of antimicrobial resistance. The present study helps to design targeted future interventions to ensure rational antimicrobial use and decrease the impact of antimicrobial resistance in Malaysia.

## Introduction

Antimicrobials have reduced the death rate due to infectious diseases for the past 70 years when they were first introduced. Whereas, inappropriate use of these antimicrobials made many micro-organisms resistant to them (1). Antimicrobial resistance (AMR) is an emerging problem throughout the world that cause increased mortality, morbidity, and cost of the treatment (2). In Europe, more than 25,000 deaths have been recorded annually due to AMR and around €1.5 billion is the cost to cope with these drug-resistant infections due to increase in the healthcare expenses and low treatment outcomes (1).

To increase the rational use of antimicrobials, the prescribing pattern of physicians should be changed (3). Antimicrobial stewardship programs (ASP) play a significant role to rationalize the use of antimicrobials in health care settings. Educating health care professionals by ASPs can decrease unnecessary prescribing of antimicrobials, cost of therapy and hospital stay (4). Health care providers are in support of ASPs because they can improve their knowledge, practice, and care of their patients more efficiently (5). Previous studies reported that physicians agreed on the excessive use of antimicrobials all over the world but denied this issue in their own health care settings (6, 7). Appropriate use of antimicrobials solely depends on the knowledge of physicians toward the antimicrobial usage guidelines which vary from country to country (3, 8). Adherence to antimicrobial guidelines by the physicians should be monitored to evaluate their prescribing behavior is the best way to increase the rational use of antimicrobials and reduce the risk of AMR (9).

Countries in South East Asia contribute substantially to inappropriate antimicrobial use which leads to higher risk of AMR (10). Many South East Asian countries collaborated with WHO to conduct national situational analyses of antimicrobial management to evaluate the use of antimicrobials in their respective countries to assess the inappropriate use of antimicrobials and educate the health care providers about the rational use of antimicrobials. Regular auditing of antimicrobials increases the rational use of antimicrobials and decreases the risk of AMR (10).

Ministry of Health (MoH) in Malaysia creates awareness about rational use of antimicrobials by publishing guidelines, however, the prescription rates of antimicrobials remain at high level (70–80%) in primary care settings and hospitals (11, 12). Some studies conducted in Malaysia reported the high rate of antimicrobial prescription are due to upper respiratory infections and acute gastroenteritis which contributes for around 49.2 and 20.5% of the antimicrobials prescription respectively (13). Therefore, it can be concluded that only publishing the guidelines alone is not enough to change the prescribing habits of health care professionals. Education of physicians regarding the published guidelines and reminders at regular intervals may have some positive impact to change their prescribing patterns and increase the rational use of antimicrobials (11, 14).

Hence, the objective of current study is to explore and compare physician's prescribing patterns and perceptions about antimicrobial use and resistance in relation to the management of common infections at a tertiary care hospital.

## Methods

By in-depth reviewing the published literature regarding antimicrobials, a semi-structured interview guide was prepared attached as a Supplementary Material (1, 3–8, 15, 16). Prior to conducting interviews, the reliability and validity of this interview guide has been evaluated to strengthen the findings of the study. Experts from School of Pharmaceutical Sciences, Universiti Sains Malaysia with substantial experience in qualitative research, reviewed the initial interview guide and validated it. Three independent physicians working as head of their departments in Hospital Pulau Pinang, Malaysia reviewed and approved all the questions in interview guide to increase the appropriateness of the interview guide. Before conducting interviews, a pilot study was carried out to improve the interview guide accordingly. Face to face interviews were conducted by recording with the physicians in hospital during office hours to assure the reliability of findings of the present study.

This study was conducted in a tertiary care hospital in Penang Island, which is a Malaysian state located in the northwest of Malaysia. By using purposive sampling technique in qualitative research, physicians registered with Malaysian Medical Council were included in this study after obtaining their written consent. The prescribing patterns and perceptions of physicians regarding antimicrobials practices in big cities could be different from those in small cities or villages.

The objectives of the study were explained to the participant physicians before starting prior to signing the consent form. Physicians had the right to withdraw from the interview at any stage of the study. Interviews were conducted in the physicians' offices during office hours. To ensure privacy of the respondents, identity of the physicians was kept anonymous. Interviews were audio recorded and then transcribed into different codes against the views of the physicians to assure the confidentiality and privacy by the principal investigator (AA). Interviews were conducted with the physicians until the saturation point has been achieved. English language was used for interviews and their duration of an interview was ~15–35 min. Physicians had the freedom to add their additional views and share experiences related to their antimicrobial practices. Saturation point had been achieved by the tenth interview; however, two more interviews were conducted to confirm saturation. All these twelve interviews were conducted from November 2019 to February 2020.

## Analysis

All recorded interviews were transcribed precisely, and the language used to write transcripts was English. To avoid grammatical errors, slight changes have been done in transcripts in the process of data extraction. Firstly, thematic content analysis was carried out independently by three researchers (AA, AHK, and HZ) in which similar views of physicians related to general questions were highlighted. Secondly, different opinions of physicians related to same research questions were compared to find out any differences between them. Emerging themes from the transcripts were identified. All the transcribed interviews were analyzed by the research team repeatedly before categorizing into the themes and sub-themes. In this way, similar themes were categorized collectively to avoid the repetition of the data.

## Results

A detailed description of all the interviewees is presented in Table 1. Out of 12 respondents, 7 were male physicians and 5 were female. Mean age of physicians were 37 ± 8 years. Following are the four major themes including sub-themes emerged from the analysis of transcribed interviews:

1. Prescribing patterns of physicians regarding antimicrobials;
2. Physician's knowledge about antimicrobials;
3. Antimicrobial Resistance (AMR);
4. Satisfaction with management of infections.

**Table 1:** Demographics of physicians.

**Sr. no**	**Gender**	**Experience (years)**	**Qualification**	**Country of graduation (Malaysia/outside Malaysia)**	**Designation in hospital**	**Area of expertise**
Phy 01	Male	5	MD	Malaysia	General Physician	Internal Medicine
Phy 02	Female	7	MRCP	Malaysia	Medical Specialist	Internal Medicine
Phy 03	Female	10	MD	Outside Malaysia	Medical Specialist	Dermatology
Phy 04	Male	4	MBBS	Malaysia	General Physician	Dermatology
Phy 05	Male	3	MBBS	Malaysia	Medical Officer	Internal Medicine
Phy 06	Male	21	MS, MBBS	Malaysia	Chest Physician	Respiratory Medicine
Phy 07	Female	6	MBBS	Malaysia	Medical Officer	Internal Medicine
Phy 08	Female	8	MBBS	Outside Malaysia	Medical Officer	Internal Medicine
Phy 09	Male	7	MD	Malaysia	Medical Officer	Internal Medicine
Phy 10	Female	26	MBBS	Malaysia	Chest Specialist	Respiratory Medicine
Phy 11	Male	16	MBBS	Malaysia	Medical Officer	Dermatology
Phy 12	Male	18	MS, MBBS	Malaysia	Consultant Dermatologist	Internal Medicine

**Theme 1:** Prescribing patterns of physicians regarding antimicrobials

**i) Decision of antimicrobial prescribing**
The majority of physicians stated that the decision to prescribe antimicrobials is solely based on the clinical condition of the patients.
“*Based on the clinical features of the patients whether they are in infected state, risk factors of the patients or they are immunocompromised, or weather is there any history of recent hospitalization.” (Phy 02)*
“*Firstly, it depends on the hospital guidelines and then go through clinically depending on the severity of the infections.” (Phy 08)*
“*By assessing the patient's condition like if a patient got fever, sign of infection, inflammation, pain and start antimicrobials.” (Phy 11)*
**ii) Difference in prescribing patterns among physicians**
Most of the physicians explained that their prescribing patterns depends on the guidelines given by the hospital and slightly different from each other due to the experience in their respective fields.
“*Yes, because of new antibiograms coming, new microorganisms and new culture sensitivity results then we adjust accordingly to what is the most predominant bug we have.” (Phy 06)*
“*Yes, I do. I think I get more experience so based on clinical symptoms then you can expect the kind of bacteria that the patient may have then you will prescribe antibiotic accordingly.” (Phy 08)*
“*Yes, based on the changes in the guidelines so it's changed according to antibiogram of the hospital as well.” (Phy 10)*
“*Yes, is ever changing because of the change of guidelines and emergence of new resistance strains.” (Phy 04)*
**iii) Prescribe antimicrobial or wait and watch approach**
Respondents included in current study said that wait and watch approach for prescribing antimicrobials depends on condition of the patients whether to start antimicrobials immediately or wait for the culture and sensitivity results.
“*Usually those are referred to our specialist clinics they are more severe cases, they are more resistance and expensive so most of them they have systemic symptoms so we most likely to start antimicrobials rather than wait and see.” (Phy 12)*
“*Most important is the clinical features how the patient is presenting, if the patient is very ill or looks like it's a bacterial infection, we won't wait.” (Phy 06)*
“*Usually this one we will see the patient's general conditions or the blood parameters then only we will decide.” (Phy 05)*
“*Normally we wait and see if there is no other worsening of the symptoms, we just treat accordingly to whatever symptoms he came in with but should there be anything while he admitted or we think he is having infection then we will start antimicrobials.” (Phy 06)*
“*We must closely monitor the condition of patient, suggest investigation and then decide what to prescribe.” (Phy 03)*
**iv) Alternatives to antimicrobials**
Interviewed physicians stated that they use alternatives to antimicrobials by examining the condition of the patients, give symptomatic treatment and suggests further investigations.
“*Symptomatic relief for example a patient is having fever we can give hydration, peptic sponging, encourage oral intake.” (Phy 08)*
“*I think if antimicrobial is not indicated then we will give symptomatic treatment.” (Phy 04)*
“*Then I will suggest that patient use those antiseptics wash and to monitor the patient and follow up more closely for the signs of infection.” (Phy 02)*

**Theme 2:** Physician's knowledge about antimicrobials

Most of the physicians have the access to the latest guidelines on antimicrobials published all over the world alongside the local guidelines by the Ministry of Health, Malaysia. Organization of CMEs by the hospital administration at regular intervals helps health care providers with latest education on antimicrobials. Detailed views of physicians regarding knowledge of antimicrobials and its related factors can be seen in Table 2.

**Table 2:** Physician's antimicrobial knowledge.

**Sr. no**	**Sub themes**	**Views of physicians**
1	Antimicrobial's information sources	“*Read a lot of journals on the web, new guidelines that's all.” (Phy 06)* *“By reading journals, familiarize myself with the local and international guidelines.” (Phy 08)* *“From recent guidelines and infectious disease stewardship.” (Phy 04)* *“I go by the clinical practice guidelines or the local hospital guidelines.” (Phy 07)*
2	Continuous medical education (CME) programs	“*Usually is CME formula in our daily practice, talked to the consultants, learning during rounds.” (Phy 01)* *“Yes, antimicrobial stewardship programs by the infectious disease unit and also hospital CME's.” (Phy 03)* *“Usually the Infectious disease unit provide us CME's for the antimicrobials.” (Phy 05)* *“I will attend CMEs, read some journals and get opinion from my specialists.” (Phy 11)* *“We follow CME and also hospital they do update their policies time to time.” (Phy 12)*
3	Availability of antimicrobial guidelines	“*Yes, we have infectious disease meetings twice a year in which they tell us about what the new organisms are coming in different parts of the hospital and then if there any new guidelines they sent to us.” (Phy 06)* *“We have booklet for the hospital guidelines, I think they have the soft copy as well that is being segregated in the WhatsApp groups.” (Phy 08)* *“Usually the hospital infectious disease unit will on and off organize course regarding antimicrobials.” (Phy 09)*

**Theme 3:** Antimicrobial Resistance (AMR)

**i) AMR being a problem**
Most of the physicians interviewed in the study admit that AMR is an emerging problem all over the world and if this problem persists it would be life threatening and may increase the cost of treatment. Moreover, it may decrease the treatment outcomes of the patients prescribed with antimicrobials.
“*Yes, there has been risk for the incidence of antimicrobial resistance over the years and I think probably due to a lot of patients who are resistant to some form of antibiotics prior to admission in the hospital.” (Phy 02)*
“*Yes, we are definitely seeing an increase in the more resistant organism specially those coming from ICU, they are so resistant sometimes that we run out of antimicrobials to use so we definitely seeing an increase in the last few years.” (Phy 06)*
“*Yes definitely, I think it happens because people are very liberal with antibiotic usage even for viral issues, they start prescribing antimicrobials and this leads to antimicrobial resistance.” (Phy 07)*
“*Yes, because some people are resistant towards antibiotics that they need to be on antimicrobial for a long period of time and sometimes patient die because of antimicrobial resistance.” (Phy 11)*
**ii) Future with AMR**
Respondents included in the present study stated that if AMR is not controlled now, it will result in very drastic circumstances in the future which may lead to higher mortality rate due to infections.
“*Currently the antimicrobial resistance is increasing in trend but I think so far it is still under control.” (Phy 02)*
“*I think it will increase mortality because there are more infections that are not treatable if AMR is an issue in the future.” (Phy 04)*
**iii) Intensity of AMR**
Physicians shared their experiences about the intensity of AMR and stated that if the burden of AMR is not controlled now, it will egregiously affect the health care system all over the world.
“*If we do not use our antimicrobial correctly, definitely the resistance will become more and more, this may become a major and serious problem in the future.” (Phy 01)*
“*So, the antimicrobial should be carefully prescribed or there will be an increase in the antimicrobial resistance in the future.” (Phy 02)*
“*I think if we are not careful, we will reach that stage where we might not have any antibiotics to prescribe, so we have to become a bit more vigilant with what we use now.” (Phy 06)*
“*It depends on doctor's practice; some doctors like to give antibiotics first due to patient's request so the antibiotic resistance is increasing.” (Phy 11)*

**Theme 4:** Satisfaction with management of infections

Many physicians admit that there are unnecessary prescriptions of antimicrobials in their health care system, but it can be reduced by regularly educating the health care providers with the latest guidelines and ASP. Educational activities for patients and the general public regarding antimicrobial usage and preventive measures should be initiated for the better control of infections. Table 3 shows the detailed views of physicians regarding their infection management satisfaction and their related factors.

**Table 3:** Physician's infection management satisfaction.

**Sr. No**	**Sub themes**	**Views of physicians**
1	Unnecessary antimicrobial prescribing	“*I think this have no answer because ill patients must start antimicrobials but if the antibiotic is started unnecessarily for example; for viral then it is not appropriate practice then it should be reduced.” (Phy 01)* *“Yes, I think antimicrobial prescription should be reduced and it can be achieved by continuous update of the physician on the latest antimicrobial brands and also if they can attend more international conferences on the antimicrobial resistance.” (Phy 02)* *“It should be reduced yes may be we need to educate our health care workers that not all fever so on requires antibiotics there are more certain to be viral so I think the prescribing physician needs to be a bit more aware when to give and when not to give antimicrobials.” (Phy 06)* *“Yes, I think need to use antimicrobials more sparingly not just by looking at one symptom, must look at the patient based on the clinical symptoms.” (Phy 08)* *“For those limited localized disease, we should be able to start treating patients with antiseptics first rather than we directly give antimicrobials.” (Phy 12)*
2	Education to patients and public	“*There should have some awareness and do not take too many types of antimicrobials without any strong source of infection and should consult a doctor for proper management.” (Phy 02)* *“We can reduce if we actively advise them to go for common vaccinations, I think that is lacking in our country specially influenza and pneumococcal vaccinations.” (Phy 06)* *“It can be reduced through education for example information regarding hand hygiene then isolation I think the infection rate will be reduced.” (Phy 09)* *“Firstly, the education of the patient's, second thing is we do not use a lot of antimicrobials.” (Phy 11)*
3	Improvement in managing infections	“*Definitely, there is a room of improvement because day by day there are more and more antimicrobial available and in different hospitals antimicrobial resistant patterns are same or going to may be different. So that's the thing that we must keep ourselves up to date, so we know about the more suitable antimicrobial in hospital.” (Phy 01)* *“I think the only way that we can help us to improve on it is that if we have more time and more closer appointment dates to monitor the condition of the patients.” (Phy 02)* *“If we have simple test like procalcitonin is one or other methods where we can actually say this is a better infection, if that magic test can come then that would definitely help us.” (Phy 06)* *“I think I need to get familiarize with the epidemiology of the infection that is going on so that I can improve my management.” (Phy 08)* *“I think my management will be improved by reading more up to date guidelines and more up to date information regarding antimicrobial resistance ways to reduce it.” (Phy 09)* *“Regular updates on the antibiotic patterns that will help us to give the most relevant, most appropriate treatment to the patients.” (Phy 12)*
4	Barriers in management of infections	“*I think for my management is still ok but may be the hospital is sometimes overcrowded with patients and this is one of the risk factors of raising the incidence of infections among the patients.” (Phy 02)* *“Yes, there are, most likely is some antimicrobials are not available and most of the antibiotics now are quite widely get over the counter.” (Phy 04)* *“Yes, there is, as a medical officer I cannot prescribe all sort of antimicrobials so even for certain infections if I think that this is the particular antimicrobial to be used, we have the limitations because we don't have access to all types of antimicrobials.” (Phy 07)* *“Yes, the availability of antibiotics and also the cost of antibiotics.” (Phy 10)* *“I think cost is the issue because antimicrobial choices are not many you can choose from and the other thing is the use of antiseptics, patient may not afford to buy.” (Phy 12)*

## Discussion

Our findings show that physicians working in a large public hospital of Malaysia were well-aware of the fact about the overuse of antimicrobials. A majority of the physicians admits that antimicrobials are sometimes unnecessary or over prescribed nationally in their country but some of them also shared their experience that in their own hospital the use of antimicrobials should be rationalized. Previous literature reported the emergence of antimicrobial resistance among their own health care system as well as over all the world as reported in present study (6, 17).

Many studies emphasize the relation of overuse of antimicrobials with the prescribing behavior of the physicians. Physicians should change their prescribing patterns of antimicrobials according to the latest guidelines for antimicrobial usage to overcome the risk of AMR which is an emerging problem all over the world (8, 18). Healthcare providers should update their knowledge in their respective fields by reading the official guidelines of their country rather than to depend on the knowledge given by representatives of different pharmaceutical industries, whose main concern is to sell their products and make profit (19). Other educational activities should be implemented in hospitals to empower the knowledge of physicians and decrease the risk of over prescribing of antimicrobials and AMR as reported in current study and previous literature (8, 9).

Many physicians in current study stated that other health care providers over prescribe antimicrobials, but their own prescribing behavior is not in a category of over prescribing, which supports the findings of previous studies (3, 7, 17). Unnecessary use and over prescribing of antimicrobials ultimately leads to AMR. Moreover, majority of the physicians included in current study believed that they can reduce the risk of AMR by reducing the use of unnecessary antimicrobials. As reported in previous studies (6, 20), many physicians agreed that updated antimicrobial knowledge is very important in their respective fields but they also need regular educational activities related to antimicrobials, feedback on their prescribing behavior and there should be an appropriate guideline on restricted agents which require a preapproval before prescribing, helping them to reduce the unnecessary prescription of antimicrobials which leads to decrease the risk of AMR.

Almost all the physicians interviewed are well-aware of the fact that AMR is not just a national problem but a global issue which is prevailing day by day. In addition, all our interviewed physicians admit that reduction in unnecessary antimicrobial usage decreases the risk of AMR in their own country as well as all over the world as compared to only 66% respondents who agreed in the study conducted by Wester et al. (15). Regular ASP conducted in the hospital of included physicians may contribute to these differences by creating more knowledge and providing updated antimicrobial guidelines for the better use of antimicrobials and reducing the risk of AMR.

Most of the physicians believed that if emergence of AMR is not controlled now, it may lead to failure of treatment and increase the therapy cost in the near future. Physicians should take advice from their seniors about the optimal use of antimicrobials because senior physicians have more knowledge about the rational use of antimicrobials due to their vast experience in their respective fields as compared to junior physicians or fresh graduates. These findings are comparable with previous studies which also reported that senior physicians are more confident for the rational use of antimicrobials as compared to junior physicians (6, 8).

The current study found that physicians have access to a wide variety of resources regarding antimicrobials for reading and updating their knowledge to improve their antimicrobial prescribing practices. A majority of the physicians emphasize regular training on antimicrobials in their hospital that provides them latest knowledge and training to change their antimicrobial prescribing behavior to promote the rational use of antimicrobials and make strategies to overcome the burden of AMR in their local health care settings. Our study also indicates the relation of junior physicians with their seniors regarding teaching and training for the better use of antimicrobials, as senior staff shared their vast knowledge with junior physicians to improve their decision making.

Physicians included in the present study also shared many useful suggestions during the interview session to enhance the rationale use of antimicrobials locally as well as globally. These includes that strict regulations should be implemented regarding the use of antimicrobials and monitor the usage of antimicrobials at regular intervals to ensure its rationale use all over the country. A majority of the participating physicians also indicated that if they have better and accurate diagnostic equipment, the excessive use of antimicrobials could be minimized. Moreover, updated knowledge of antimicrobials and regular antimicrobial stewardship programmes in the hospital reduced the overuse of antimicrobials. Physicians appreciate the regular availability of local guidelines by the hospital management which provides updated knowledge which is consistent with an Australian survey (5). Physicians knowledge could be improved if hospital pharmacists had a heightened role in educating as well as monitoring antimicrobial utilization, which is implemented in many countries of the world. Developing ASP and implementing them is also included in the role of hospital pharmacists (21).

There are few limitations in our study. Firstly, our study was conducted in one public sector hospital, so the generalizability of the results must be viewed with caution. Secondly, physicians who do not wish to take part in our study may have different views and opinions regarding the use of antimicrobials and antimicrobial resistance, which may lead to unmeasured bias. Nevertheless, our comprehensive methodology and results investigate different aspects of all the themes which can reduce this bias. Thirdly, there is a possibility that physicians gave socially accepted answers but not their true opinions. This issue was addressed by assuring the physicians about the privacy of their opinions. Despite of these above said limitations, the objectives of current study have been achieved, as we identified different factors involved in prescribing behavior and perceptions of physicians regarding the use of antimicrobials and risk of antimicrobial resistance in their own health care system and all over the world.

## Conclusion

Physicians working in included health care setting were well-aware of the problems of unnecessary usage of antimicrobials and different factors involved in the emergence of antimicrobial resistance and they strongly believed that regular efforts may decrease the impact of these problems. Our study compares different views of physicians regarding their practices, which may be used to design targeted interventions in future. Ongoing educational activities should continue to update the physician's knowledge and latest updates on antimicrobials, which helps to decrease the risk of antimicrobial resistance which is an emerging problem throughout the world.

## Data Availability Statement

The raw data supporting the conclusions of this article will be made available by the authors, without undue reservation.

## Ethics Statement

The studies involving human participants were reviewed and approved by Current Study was conducted after the approval from National Institute of Health and Medical Research and Ethics Committee, Malaysia (NMRR-19-1037-46721). The patients/participants provided their written informed consent to participate in this study.

## Author Contributions

AA conceptualized and designed the study, conducted the statistical analyses, interpreted the data, and drafted the manuscript. AK, HZ, and MA revised the manuscript for intellectual content, read, and approved the final version of the manuscript. IA and LM helped in conducted the interviews and supervised the drafting of the manuscript, supported in interpreting the data, and revised the manuscript for intellectual content. All authors read and approved the final manuscript.

## Conflict of Interest

The authors declare that the research was conducted in the absence of any commercial or financial relationships that could be construed as a potential conflict of interest.
