# Supplementary material for: Physicians' Perspective on Prescribing Patterns and Knowledge on Antimicrobial Use and Resistance in Penang, Malaysia: A Qualitative Study
Source: Front Public Health. 2020 Nov 25;8:601961. doi: 10.3389/fpubh.2020.601961 (PMC7724042; doi:10.3389/fpubh.2020.601961)
Supplement: Supplementary file 1 [file Table_1.DOCX]

**Interview Guide:**

**Prescribing Patterns**

1. In your daily routine, how many patients (approx.) you examined in a day?
2. How you make decision for prescribing antimicrobials in your hospital?

How you see your own antimicrobial prescribing patterns in relation to others?

1. Do you think your prescribing practices have changed over time? In what way and why?
2. How do you decide exactly which type of antimicrobial to prescribe?

What factors make you more likely to prescribe an antimicrobial vs watch and wait approach?

Signs and symptoms of patients, severity of illness.

1. If you decide not to prescribe antimicrobial what are the alternatives you suggest?

**Antimicrobials**

1. **Sources of information about antimicrobials**
2. How do you keep up to date yourself on new information regarding antimicrobials?

Prompts: GP colleagues, journals, medical representatives, magazines, national or international campaigns?

1. Is education on antimicrobials available? In what form?
2. **Antimicrobial Resistance (AMR)**
3. Do you think AMR is a problem in your practice? Can you tell me more about this?
4. Do you think AMR is an upcoming problem for the country as a whole?
5. How do you see the intensity of this problem in the future?

**Interventions**

1. Do you think that number of patients visiting for infections could be reduced? If yes, how could this be achieved?

Prompts: interventions, antibiotic brands in market should be reduced, review of national guidelines.

1. Do you think that number of antibiotics prescribed to patients should be reduced? If yes, how could this be achieved?

Prompts: information for physicians, information for patients, national guidelines review, antibiotic brands limitation.

1. Are you satisfied with the way you manage infections?

Follow up:

- Do you think your management could be improved? What might help you most to improve your practice?
- Are there any barriers for changing your management? If yes, what are they?
